# Real-time analysis of quantum dot labeled single porcine epidemic diarrhea virus moving along the microtubules using single particle tracking

**DOI:** 10.1038/s41598-018-37789-9

**Published:** 2019-02-04

**Authors:** Wei Hou, Yangyang Li, Wenjie Kang, Xin Wang, Xuping Wu, Shouyu Wang, Fei Liu

**Affiliations:** 1 Joint International Research Laboratory of Animal Health and Food Safety, Single Molecule Nanometry Laboratory (Sinmolab) & Key Laboratory of Animal Physiology and Biochemistry, Nanjing Agricultural University, Nanjing, 210095 China; 2 The Second Hospital of Nanjing Affiliated to Southeast University, Nanjing, 210003 China; 3 Computational Optics Laboratory, School of Science, Jiangnan University, Wuxi, 214122 China

## Abstract

In order to study the infection mechanism of porcine epidemic diarrhea virus (PEDV), which causes porcine epidemic diarrhea, a highly contagious enteric disease, we combined quantum dot labeled method, which could hold intact infectivity of the labeled viruses to the largest extent, with the single particle tracking technique to dynamically and globally visualize the transport behaviors of PEDVs in live Vero cells. Our results were the first time to uncover the dynamic characteristics of PEDVs moving along the microtubules in the host cells. It is found that PEDVs kept restricted motion mode with a relatively stable speed in the cell membrane region; while performed a slow-fast-slow velocity pattern with different motion modes in the cell cytoplasm region and near the microtubule organizing center region. In addition, the return movements of small amount of PEDVs were also observed in the live cells. Collectively, our work is crucial for understanding the movement mechanisms of PEDV in the live cells, and the proposed work also provided important references for further analysis and study on the infection mechanism of PEDVs.

## Introduction

Porcine epidemic diarrhea virus (PEDV), a member of the group I coronaviruses, is an enveloped RNA virus with a single, positive-stranded genome of about 30 kb. It was initially reported in England in 1971, since then it has been identified in many swine-producing countries in Europe and Asia, notably in Belgium, Italy, South Korea, Japan and China. PEDV can cause porcine epidemic diarrhea (PED), a highly contagious enteric disease characterized by vomiting, acute watery diarrhea, and in particular, a high mortality rate in suckling piglets, resulting in substantial economic loss. The PED outbreak in 2017 was a mixed infection caused by various genotypes of PEDV^1^, putting PED in a new situation. New drugs and vaccines are required to fight the PEDV infection, however, the mechanism of PEDV infection of host cells still remains unclear, limiting the development of new vaccines and effective PED treatments.

In order to study the PEDV infection mechanism, several works have been proposed as to reveal the endocytic pathways^2,3^ and the receptors^4–6^. However, the intracellular transport of PEDV was uninvestigated. It is known that microtubule (MT) is a component of cytoskeleton and essential for the intracellular transport of cargoes based on molecular motors^7–9^. Dynein and kinesin are both intracellular motor proteins that move unidirectionally in opposite directions along MTs, which may lead to the complex movements of the cargoes along the MTs in live cells^10–12^. As obligate parasites, viruses such as human immunodeficiency virus (HIV)^13^ and parvoviruses^14,15^ require MTs during cell entry for efficient nuclear targeting, either for cytosolic transport of naked viral particles or for transport inside vesicles. During assembly and egress, viruses such as HIV^16^ and African swine fever virus^17^ also need MTs for trafficking inside exocytic vesicles or for cytosolic transport of capsids and nucleoprotein particles to the budding compartment. The MT functions throughout the whole virus infection cycle. However, there are still no reports about the interactions of PEDVs with MTs in host cells. In order to clearly reveal the PEDV motion along the MTs in the live cells, tracking the interactions between the PEDV and the MTs in the live cells is required, which is not only useful for monitoring the PEDV invasion process, but also significant for in-depth understanding of the mechanisms of PEDV infection.

Compared to the traditional methods, such as cell fractionation, electron microscopy and immunofluorescence assay, which only focus on the static observations, single particle tracking is a better choice, since it can provide the dynamic processes or short-lived events during virus invasion and infection in the live host cells directly and authentically^18,19^. Furthermore, single particle tracking can provide important references for further virus infection mechanism research. For example, poliovirus (PV) was originally found to enter cells via clathrin-mediated endocytosis^20^. By using single particle tracking, it was further showed that PV released from the vesicles or tightly sealed membrane invaginations located within 100–200 nm of the plasma membrane highly efficiently and rapidly^21^. Therefore, considering the advantages of single particle tracking, it was adopted here to observe the PEDVs moving along the MTs in the live cells directly. However, it is worth noting that in order to track the PEDVs along the MTs, virus labeling is required, and various labeling methods have been designed including genetic engineering, physical incorporation, and bioconjugation. Genetic engineering can label viruses at a well-defined position by fusing fluorescent proteins (FPs) to certain domains of virus proteins or viral nucleic acids, however, this method has great challenges due to the complicated processes, technical barriers and even the inactivation of labeled viruses^22^. Physical incorporation usually utilizes lipophilic fluorescence reagents such as DiD, Rhodamine-18 and their analogues to enter into the lipid membrane of the envelope of viruses, but it is compromised for low specificity^13,18,23–29^. Bioconjugation labels the virus by using the chemical reaction between the chemical groups of viruses and fluorophores^30^ while chemical modifications usually have a low efficiency and influence the activity of the viruses^31^. Therefore, optimized labeling tactic is still required for long time tracking of PEDV moving along the MTs in the live host cells.

**Figure 1:**
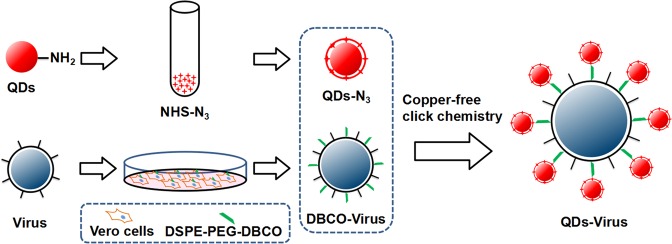
Schematic illustration of virus labeling via click chemistry and phospholipid analogues. The DSPE-PEG-DBCO modified viruses were generated through virus assembling and aided by DSPE-PEG-DBCO modified host cells. QD-N_3_ was generated by adding the azidobutyric acid NHS eater (NHS-N_3_) to the QD-NH_2_. The viruses were then labeled by QD-N_3_ via copper-free click chemistry with DBCO.

In order to realize real time PEDV tracking, as well as to less influence PEDV, we labeled PEDV with quantum dots (QDs) through copper-free click chemistry using 1,2-distearoyl-sn-glycero-3-phosphoethanolamine-N-[dibenzocyclooctyl (polyethylene glycol)-2000] (DSPE-PEG-DBCO) fed cells directly as shown in Fig. 1. QDs were chosen since they are 10–100 times brighter and 100–1000 times more photostable than organic dyes and FPs, thus making them ideal for long-time tracking of viruses^32,33^. Compared to modified viruses by utilizing the streptavidin-conjugated QDs^34^, which significantly increased the size of viruses^35^ and decreased the infectivity of labeled viruses, our optimized labeling strategy less influenced the virus dynamics and hardly affected PEDV infectivity. With the optimized labeling technique, it is the first time, to our best knowledge, long time tracking of massive QD modified PEDVs along the MTs were successfully realized in real time, the statistical results show that PEDV had specific motion modes in different regions when they traveled along the MTs in the live Vero cells. Besides, small amount of PEDVs also experienced a return movement in live cells occasionally. Combining with the real time single particle tracking technique and the optimized labeling method, the dynamics of the PEDVs moving along the MTs were observed directly. In addition, with the quantitative analysis on trajectory, velocity and mean square displacement, both the motion velocity and motion mode of PEDVs were extracted, providing important references for further study on the PEDV infection mechanism, and the development of new vaccines or effective PED treatments.

## Materials and Methods

### Propagation, Purification and Labeling of virus

Vero cells were first cultured in Dulbecco’s modified Eagle’s medium (DMEM, Gibco) supplemented with 10% fetal bovine serum (FBS, Gibco) and maintained at 37 °C with 5% CO_2_. DBCO modified Vero cells were produced by DSPE-PEG-DBCO (Avanti) with various concentrations of 2.5, 5, 10, 20 and 30 µg/mL at 37 °C with 5% CO_2_. Then, PEDV strain CV777, a commercially attenuated vaccine virus, was propagated in Vero cell monolayer. The cells were washed three times with serum-free DMEM, and cultured with 0.3% tryptose phosphate broth (TPB, Sigma) and 3 µg/mL tripsin (Sigma) in the DMEM for 72 h at 37 °C with 5% CO_2_. Finally, DBCO modified viruses were propagated in DBCO modified Vero cells in the same condition. The cell debris was removed by centrifugation at 850 g for 10 min after freezing and thawing twice, and the viruses were purified by a 10–60% gradient of sucrose at 100,000 g for 2 h at 4 °C.

QD-N_3_ was produced by adding the azidobutyric acid NHS eater (Lumiprobe Corporation, US) (NHS-N_3_) to the QD-NH_2_ (Wuhan Jiayuan Quantum Dots Co., Ltd., China) in the 0.1 M NaHCO_3_-Na_2_CO_3_ buffer solution (pH = 7–9) and stirred at 25 °C for 1 h. Then QD modified viruses were obtained by mixing the virus solution and QD-N_3_ for 1 h at 25 °C. Unbound QD-N_3_ was removed by gel filtration on a NAP-10 column (GE Healthcare).

### MTT Assay

Vero cells were seeded in a 96-well plate with different concentrations of DSPE-PEG-DBCO (2.5, 5, 10, 20 and 30 µg/mL). Then, 10 µL 3-(4,5-dimethyl-2-thiazolyl)-2,5-diphenyl-2-H-tetrazolium bromide (MTT, Aladdin) was added to each concentration at different time (6, 12, 24, 36, 48 and 72 h), respectively. After incubation with MTT for 4 h, the supernatant was removed, and 150 µL dimethyl sulfoxide (DMSO, Sigma) was added. The UV absorbance was measured at 490 nm.

### Virus Titer Assays

The virus titer was quantified by 50% Tissue Culture Infective Dose (TCID_50_). Vero cells were cultured in the 96-well plates in DMEM supplemented with 10% FBS. Then, the cells were infected by the virus samples, which were serially diluted by 10-fold ranging from 10^−1^ to 10^−8^ in DMEM with 0.3% TPB and 3 µg/mL tripsin. The infected cells were cultured in an incubator at 37 °C with 5% CO_2_ for 24 h. Two ways according to indirect immunofluorescence and cytopathic effect can be adopted for virus titer evaluation. Using the indirect immunofluorescence based method, the infected cells in the 96-well plates were fixed with 4% (w/v) paraformaldehyde for 20 min, and the cells were incubated with mouse anti-N protein monoclonal antibody against PEDV N protein at 37 °C for 1 h and washed with 1 × PBS buffer (137 mM NaCl, 2.7 mM KCl, 10 mM Na_2_HPO_4_, 2 mM KH_2_PO_4_) to remove excess antibodies, followed by incubation with FITC 488 conjugated goat-antimouse IgG (Thermo) at 37 °C for 1 h, and wash by 1 × PBS buffer to remove excess antibodies. The number of fluorescent cells was counted by indirect immunofluorescence. Besides, the virus titer could also be quantified by observing and counting the number of cytopathic effect (CPE) on Vero cells in the 96-well plates. The TCID_50_ was calculated based on the Reed and Muench method^36^.

The one-step growth curves of the wild-type virus, DBCO modified virus or QD modified virus were obtained by measuring the TCID_50_ of the viruses on normal Vero cells at 6, 12, 24, 36, 48 and 72 h, respectively^37^.

### Fluorescence Colocalization Assay

Vero cells were cultured in DMEM with 10% FBS and supplemented with or without DSPE-PEG-DBCO (DBCO) to a monolayer at 37 °C with 5% CO_2_. Then, the cells were washed by 1 × PBS buffer to remove excess DBCO. Finally, QD-N_3_ was added to the cells at 37 °C for 1 h, and cells were washed by 1 × PBS buffer to remove excess QD-N_3_ and fixed with 4% paraformaldehyde. The prepared samples were observed with a fluorescent microscope (Nikon A1 Plus si STORM).

Both wild-type and DBCO labeled viruses were first incubated with QD-N_3_ for 1 h at room temperature, respectively. Unreacted QD-N_3_ was removed by gel filtration on a NAP-10 column. Then, Vero cells were incubated with the as-prepared viruses at 4 °C for 30 min to allow attachment or continued to be incubated for a proper time at 37 °C with 5% CO_2_. Thereafter, the infected cells were washed with 1 × PBS buffer and fixed by 4% (w/v) paraformaldehyde for 20 min at −20 °C, followed by incubation with mouse monoclonal antibody against N protein of PEDV at 37 °C for 1 h and wash by 1 × PBS buffer to remove excess antibodies. Next, the cells were incubated with FITC labeled goat anti-mouse IgG polyclonal antibody at 37 °C for 1 h and washed with 1 × PBS buffer to remove excess antibodies. The nuclei of the cells were stained with 1 μg/mL DAPI solution for 10 min. Finally, the prepared samples were observed with a fluorescence microscope (Nikon A1 Plus si STORM), and the colocalization coefficient of the fluorescent signals was analyzed. QD was excited by a laser with the wavelength of 561 nm, and the emission was detected at 550–650 nm; DAPI was excited by a laser with the wavelength of 405 nm, and the emission was detected at 420–500 nm; and FITC was excited by a laser with the wavelength of 488 nm, and the emission was detected at 500–600 nm.

### Fluorescence Imaging and Analysis

Vero cells were incubated in a 35 mm confocal dish with a proper density, and then the cells were transiently transfected with plasmids encoding EGFP-microtubule using Lipofectamine 3000 reagents (Invitrogen). After 24 h, the cells were infected with QD labeled PEDVs for 30 min at 4 °C for virus attachment, next the PEDV movements in the live cells were observed and recorded with a fluorescence microscope (Nikon A1 Plus si STORM) at 37 °C in a 5% CO_2_ environment. Image series were recorded with the frame interval of 4 s^38^. The trajectory and velocity of the labeled viruses were analyzed. Besides, the mean square displacement (MSD) was also computed. The MSD at time interval Δt was the average of all squared displacements throughout the virus trajectory during association with MTs. Different motion modes of viruses were determined by fitting the dependence of MSD against time with the functions as <r^2^> = 4DΔt (normal diffusion), <r^2^> = 4DΔt^α^ (α < 1, restricted diffusion) and <r^2^> = 4DΔt + (VΔt)^2^ (directed diffusion), where D represents the diffusion coefficient, V represents the mean velocity, and Δt represents the time interval.

## Results and Discussion

### Evaluation on DSPE-PEG-DBCO modified Vero cells and DSPE-PEG-DBCO or QD modified PEDVs

**Figure 2:**
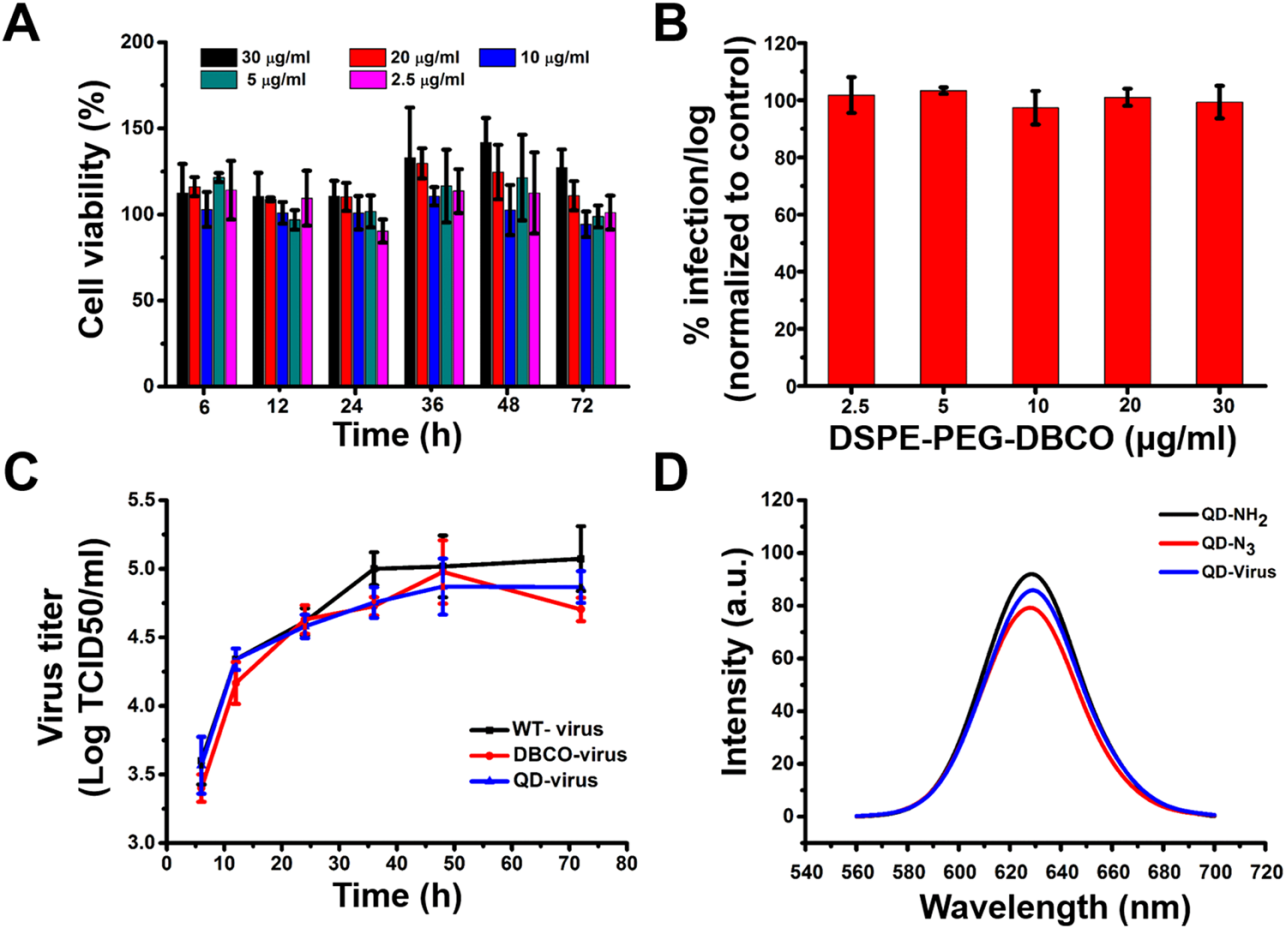
Characterization of modified Vero cells and PEDVs. (**A**) MTT assay of Vero cells cultured at different concentrations of DSPE-PEG-DBCO for different hours. Each data point represents mean ± standard deviation (n = 9, P < 0.05) and was normalized to the Vero cells cultured without DSPE-PEG-DBCO in DMEM at the same time. (**B**) Titer of virus labeled with different concentrations of DBCO. Each data point represents mean ± standard deviation (n = 9, P < 0.05). (**C**) One-step growth curves of wild-type virus (WT-virus), DBCO labeled virus (DBCO-virus), and QD modified virus (QD-virus). Each data point represents mean ± standard deviation (n = 9, P < 0.05). (**D**) The emission spectra of QD-NH_2_ (black), QD-N_3_ (red) and QD-virus (blue).

First, the cytotoxicity of DSPE-PEG-DBCO treatment on the host Vero cells was analyzed. As shown in Fig. 2(A), Vero cells maintained similar viability in DSPE-PEG-DBCO to those in DMEM, even with DSPE-PEG-DBCO concentration up to 30 µg/mL for at least 72 h, therefore, DSPE-PEG-DBCO does not hinder cell viability. Next, the activity of modified viruses was evaluated via virus titer. As shown in Fig. 2(B), the labeled viruses generated from various concentrations of DSPE-PEG-DBCO maintained similar infectivity compared to the wild-type viruses. Considering the solubility of DSPE-PEG-DBCO is around 30 µg/mL, we chose its concentration as 20 µg/mL for cell and virus modification. Additionally, the characteristic of the QD-N_3_ was also verified by a colocalization assay. As shown in Figure S1, the QDs and colocalization signals could only be detected in the QD-N_3_ incubated DBCO modified virus (DBCO-virus) while no QDs or colocalization signals were detected with the QD-NH_2_ or NHS-N_3_ incubated DBCO-virus, indicating that the QD-N_3_ was successfully generated by the reactions of QD-NH_2_ and NHS-N_3_. Besides, the activities of DBCO modified virus and QD modified virus (QD-virus) were further evaluated by measuring the one-step growth kinetics. As shown in Fig. 2(C), the growth kinetics of DBCO- or QD-viruses was similar to that of the wild-type ones, indicating that DBCO- or QD-viruses maintained the same infectivity as the wild type. Finally, the emission spectra of the QD-viruses were measured as shown in Fig. 2(D), the QD still maintained its emission features after connecting with viruses.

### Specificity of the QD-N_3_ labeling on the DBCO-installed Vero cells and viruses

**Figure 3:**
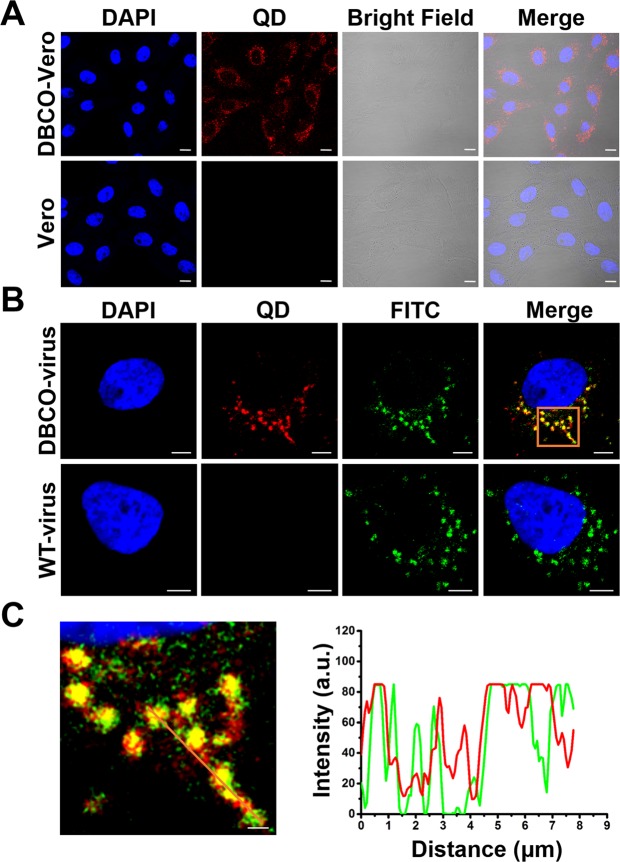
Specificity of the QD-N_3_ labeling on the DBCO-installed Vero cells and virus. (**A**) Multimode imaging of Vero cells with or without DSPE-PEG-DBCO labeling. Scale bars, 10 µm. (**B**) Fluorescence imaging of DBCO-viruses and wild type viruses (WT-virus) in Vero cells, including fluorescence images of the QDs (red), immunofluorescent signals of viruses (green), DAPI (blue), and their merged images (yellow). Scale bars indicate 5 µm of regular views. (**C**) The distributions of the signals from QDs (red) and immunofluorescent signals of viruses (green) along the line. Scale bar, 1 µm.

To evaluate whether the Vero cells could be labeled with DBCO, QD-N_3_ was added to the Vero cells cultured with or without DSPE-PEG-DBCO (DBCO) as shown in Fig. 3(A), the QD signals could only be detected in the DBCO cultured Vero cells, proving that the Vero cells could be labeled with DBCO. The results also demonstrated the reaction between QD-N_3_ and the DBCO. Besides, in order to determine that the QD-N_3_ modified viruses were specifically produced by the chemical bond formed between the QD-N_3_ and the DBCO labeled viruses, a colocalization assay was used to evaluate the specific labeling of QD-N_3_ to the viruses. As an example shown in Fig. 3(B), 75.6% ± 4.9% of the immunofluorescence signals were colocalized with QD signals, suggesting that most of the virions budding from the DBCO cultured cells could be modified with DBCO. Besides, 81.7% ± 3.5% of the QD signals were colocalized with immunofluorescence signals while no QDs or colocalization signals were detected with wild-type viruses, illustrating that the QDs were adequate and specific for displaying the PEDVs with negligible interference from nonspecific binding. Therefore, the observed QDs signals were mostly from the labeled viruses, other than from the free QD-N_3_. Furthermore, the line profile of the enlarged region form Fig. 3(B) showed that nearly all the intensity peaks of the QD signals coincide the intensity peaks of the immunofluorescence signals as shown in Fig. 3(C). Both the data in Fig. 3(B, C) demonstrates that QDs colocalized with the viruses with high specificity. It is worth noting that the images shown in Fig. 3 were only representatives from 60 fields of views, and the data were statistically extracted from all the fields of view.

### Real time single particle tracking of PEDVs along the MTs in Vero cells

**Figure 4:**
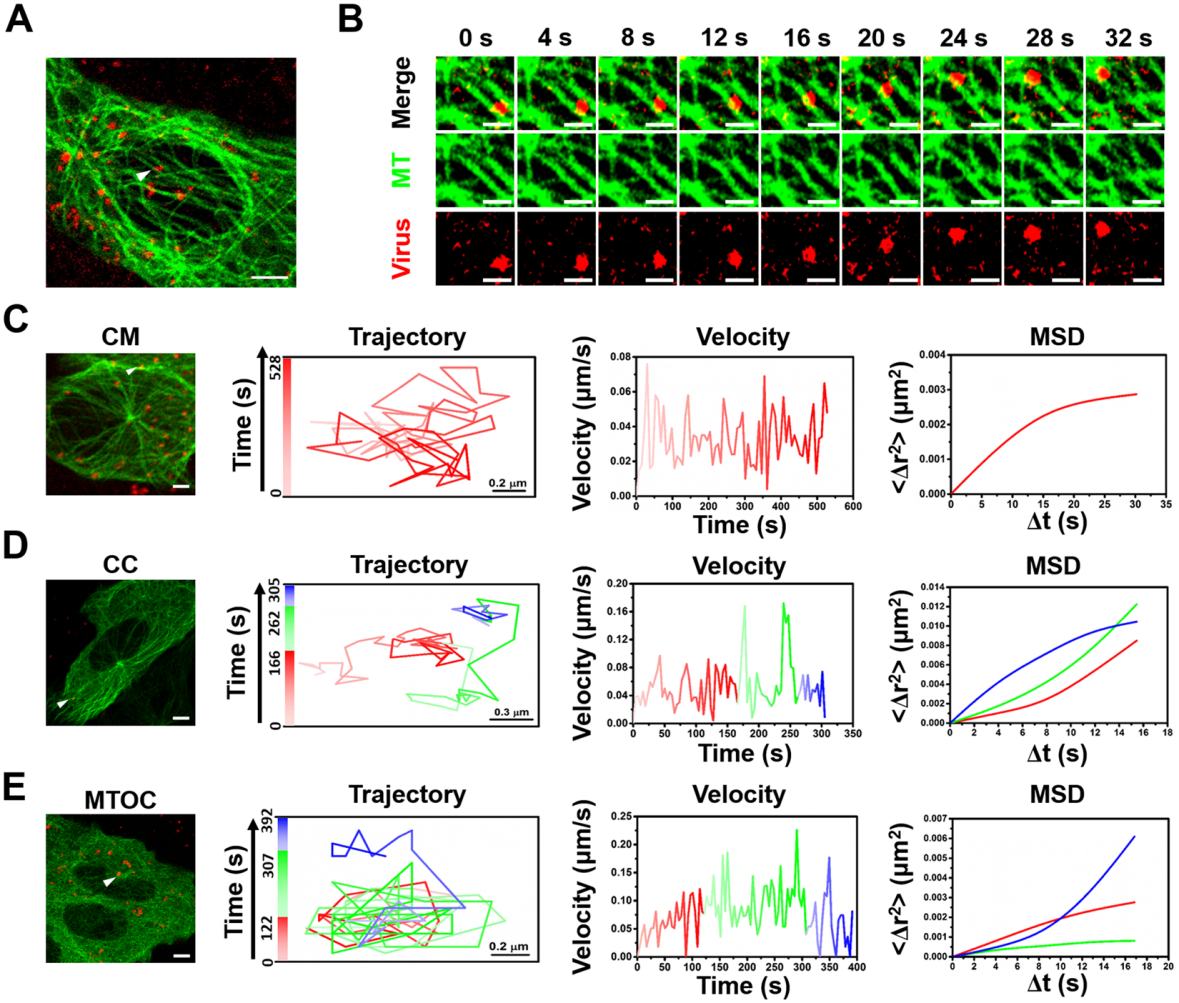
Real time tracking of PEDVs along the MTs in the Vero cells. (**A**) Captured image of QD-viruses (red) colocalized with the MT (green). Scale bar, 5 μm. (**B**) Time-lapsed image of QD-virus along the MT selected from the white arrow in (**A**). Scale bars, 1 μm. Trajectory, velocity and fitted MSD in (**C**) CM, (**D**) CC and (**E**) MTOC regions, respectively. The white bar in (**C–E**) indicates 5 μm.

After generation of QD modified PEDVs, the motions of PEDVs along MTs in Vero cells were tracked in real time with a fluorescence microscope. Totally 60 effective QD modified PEDVs moving along MTs from 20 cells were observed and measured, and their trajectories, velocities and MSD were all analyzed. And we unexpectedly found that the behavior of movements of PEDV traveling along the MTs was diverse. In addition, the dynamics of the movements along the MTs (85%, 51/60 PEDVs in 20 cells) were characterized according to the position of the interaction between the virus and MTs: the near-cell membrane (CM) region^39^ (within 1 μm of the cell membrane); the middle-cell cytoplasm (CC) region; the near-microtubule organizing center (MTOC) region (within 5 μm of the MTOC), which is a distinct region that is responsible for attaching and organizing the microtubules in living cells^40^ and is also the site of microtubule nucleation^32,41^. The fast or slow speed was defined by calculating the average speed from instantaneous velocities of 9 continuous frames. The threshold for fast or slow speed interval determination was defined as 75% of the peak value of the average velocity. If the average speed was under the threshold, we defined the movement as slow, otherwise we defined the movement as fast. Figure 4(A, B) list an example of single QD modified PEDV (red) moving along the EGFP-tagged MTs (green) (Video S1 and S2 in the Supplementary Information). According to the real time tracking, it indicates that the PEDV dynamics in the CM, CC and MTOC regions were different. When the PEDV was first located at the CM region (Video S3 in the Supplementary Information), its velocity was relatively stable with an average speed of 0.03 μm/s in Fig. 4(C), besides, the MSD was fitted by the restricted motion mode with D of 0.005 μm^2^/s (R^2^ = 0.99). Then, when the PEDV moved to the CC region in Fig. 4(D), its average speed was primarily maintained as the average speed of 0.05 μm/s similar to that in the CM region, while its motion was changed to the directional diffusion motion mode with D of 0.037 μm^2^/s (R^2^ = 0.99). With the same directional diffusion motion mode with D of 0.009 μm^2^/s (R^2^ = 0.99), the PEDV speed was next accelerated to the average speed of 0.08 μm/s. At last, the PEDV decelerated in the restricted motion mode with average speed of 0.05 μm/s and D of 0.00032 μm^2^/s (R^2^ = 0.99), respectively. The analysis indicates that the PEDV experienced a slow-fast-slow process during the motion along the MTs within the CC region (Video S4 in the Supplementary Information). Finally, when the PEDV reached the MTOC region, it also experienced a slow-fast-slow process as shown in Fig. 4(E). The PEDV primarily kept relatively slow motion in the restricted motion mode with the average velocity and D of 0.06 μm/s and 0.014 μm^2^/s (R^2^ = 0.98), respectively. Afterwards, it accelerated to 0.1 μm/s but still with the restricted motion mode with D of 0.008 μm^2^/s (R^2^ = 0.95). At last, the PEDV decelerated to an average velocity of 0.06 μm/s but in the directional diffusion motion mode with D of 0.013 μm^2^/s (R^2^ = 0.99) (Video S5 in the Supplementary Information).

**Figure 5:**
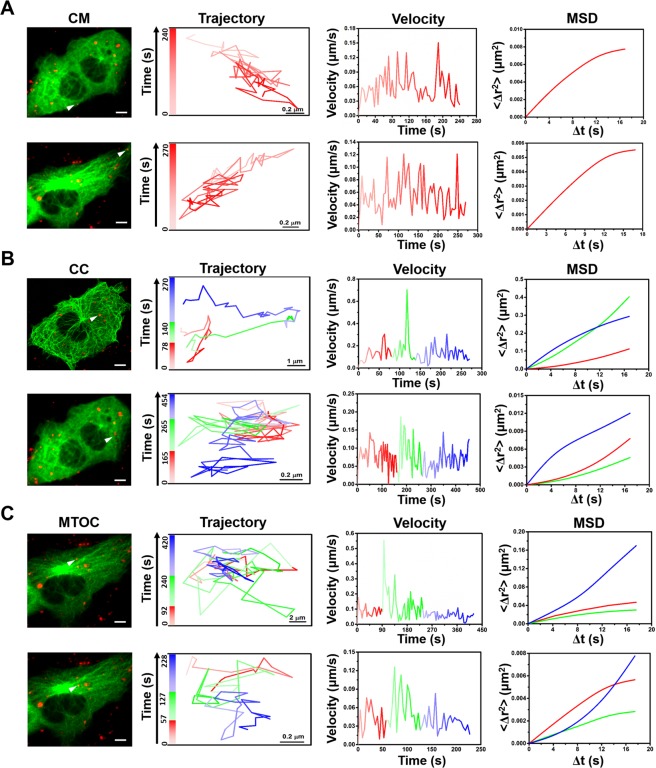
Real time tracking of PEDVs along the MTs in Vero cells. Captured image of QD-virus (red) colocalized with the MT (green), trajectory, velocity and fitted MSD in (**A**) CM, (**B**) CC and (**C**) MTOC regions, respectively. The white bars in (**A–C**) indicate 5 μm.

**Figure 6:**
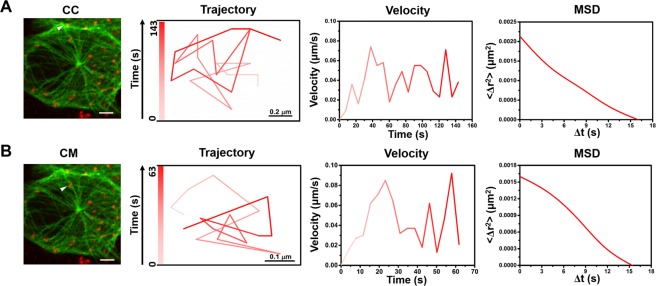
Return motion of PEDVs along the MTs in Vero cells. Captured image of QD-virus (red) colocalized with the MT (green), trajectory, velocity and fitted MSD in (**A**) and (**B**), respectively. The white bar in (**A, B**) indicates 5 μm.

Besides the PEDV shown in Fig. 4, most of the PEDVs (85%, 51/60 PEDVs in 20 cells), which are moving along MTs, also performed similar motions as relatively stable movements in the CM region and slow-fast-slow motion patterns in both CC and MTOC regions as shown in Fig. 5; besides, the motion modes presented in Fig. 5 were also the same as those in Fig. 4. During the PEDV motion in the CM region, the average velocity of the PEDVs was ~0.05 μm/s and all in restricted motion mode as two represented examples listed in Fig. 5(A). When the PEDV was in the CC or MTOC region, different PEDVs moved in diverse velocities, while they all followed a slow-fast-slow motion pattern with a directional-directional-restricted diffusion motion mode or a restricted-restricted-directional diffusion motion mode, respectively, as the two represented examples shown in Fig. 5(B, C). In addition, return movements of small amount of PEDVs (15%, 9/60 PEDVs in 20 cells) were also observed in Vero cells as shown in Fig. 6(A, B), both with the average velocity of ~0.04 μm/s (Videos S6 and S7 in the Supplementary Information). However, because the percentage of the PEDVs showing return movements was small (15%), therefore most PEDVs (85%) invaded the Vero cells in the specific motions described in Figs 4 and 5.

**Figure 7:**
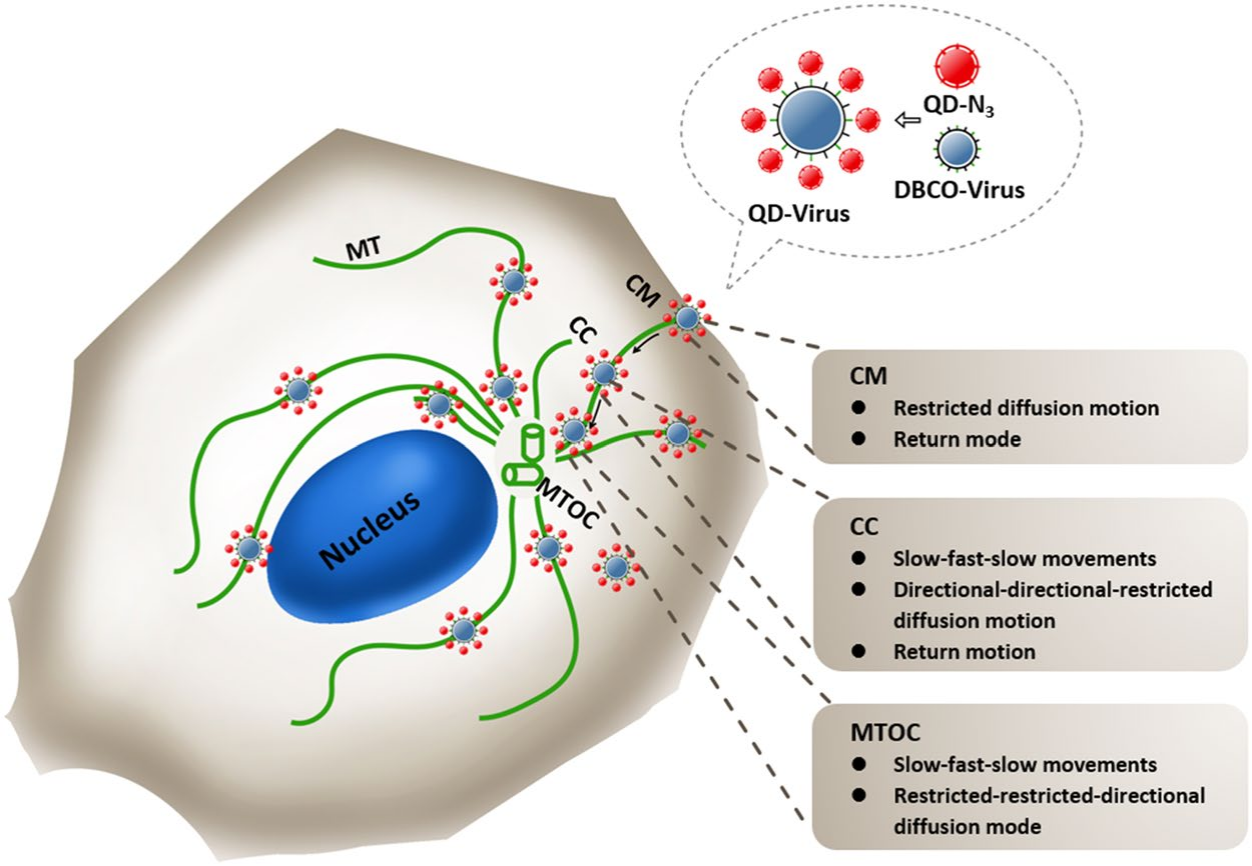
Scheme of PEDVs moving along the MTs in the Vero cell.

According to the real time tracking of PEDVs moving along the MTs in Vero cells, it shows that most PEDVs had specific infection motions as shown in the schematic diagram of Fig. 7. First, in the CM region, PEDVs performed a restricted motion mode, which is similar to that of influenza virus (IV)^42^, pseudorabies virus (Prv)^43^ and human respiratory syncytial virus (RSV)^44^ when entering the host cells. It is known that viruses exploit specific receptors to identify and infect target cells, however, these receptors are often rare or distributed in regions that are not readily accessible to incoming virions. Therefore, many viruses first bind to relatively non-specific attachment factors and migrate along the cell surface to locate specific receptor(s)^45^. Then, specific virus-receptor interactions activate signaling cascades, guiding the virus into endocytic pathways and/or triggering conformational changes in the virus envelope for entering the host cells. Therefore, entry is a rate-limiting step often in slow speed for viral infection^46^. Next, in the CC region, different from IV^25^ and Prv^43^ which showed the directional motion mode with no obvious changes in the speed, the movement of PEDVs was more diverse showing the directional-directional-restricted motion mode with slow-fast-slow speed pattern. During this period, viruses need to travel along cytoskeleton to reach a specific site for genome release after entering the cell, or travel inside endocytic or exocytic vesicles^8^. During viral transport in the cytoplasm viral proteins interact directly with dynein^47^ or kinesin^48^ motors. Since viruses widely exploit diverse cellular mechanisms for transport, the virus motion modes and the movement speeds may be more complicated during this period for PEDV. Finally, in the MTOC region, PEDVs also performed more diverse movement than that of IV by showing the restricted-restricted-directional motion mode, while IV showed either the restricted motion mode^49^ or the directional motion mode^50^ as reported in the literatures. It is most likely because near MTOC^51^ the MTs often have diversified structures^52^ making the virus movements more complicated there. Although return movements of small amount of PEDVs were also observed in Vero cells, most PEDVs still invaded into the Vero cells along MTs in specific motions. With the optimized labeling strategy and the real time single particle tracking technique, it is believed that this work established the method for direct observation on the PEDV invasion, revealed the dynamic and diverse behavior of PEDV traveling along the MTs and proposed references for further analysis on the infection mechanism of PEDVs.

## Conclusions

In order to study the mechanisms of PEDV infection, this paper provided real time tracking of the PEDVs moving along the MTs in live host Vero cells combining with the optimized labeling method and the real time single particle tracking. To label the PEDVs, we introduced DSPE-PEG-DBCO to the viral envelope via virus propagation in the DSPE-PEG-DBCO cultured Vero cells. The DSPE-PEG-DBCO has little cytotoxicity to the Vero cells, moreover, this strategy does not affect the activity or production of the viruses. After labeled with QDs by copper-free click chemistry reaction specifically, these labeled viruses can be used for single virus tracking to analyze the dynamics of the PEDV infection. Using real time single particle tracking, the infection motions of 60 PEDVs along the MTs in the host Vero cells were recorded and analyzed. It shows that most PEDVs had specific motion along the MTs: in the CM region, PEDVs performed restricted motion mode with relatively stable speed; while in the CC and MTOC regions, the velocity of PEDVs both followed the slow-fast-slow pattern, but in different motion modes. Though return movements of small amount of PEDVs were also observed in Vero cells, most PEDVs still invaded the Vero cells along MTs in the specific motions. According to the optimized labeling method as well as the detailed analysis on the PEDV motion along the MTs via single particle tracking, it is believed that this work provides direct PEDV infection model, which proposes important references for further study on the PEDV infection mechanism, and promotes the development of new vaccines and effective PED treatments.

## Supplementary information

Real-time analysis of quantum dot labeled single porcine epidemic diarrhea virus moving along the microtubules using single particle tracking

Video S1

Video S2

Video S3

Video S4

Video S5

Video S6

Video S7

## References

[1] Diep NV, Norimine J, Sueyoshi M, Lan NT, Yamaguchi R (2017). Novel porcine epidemic diarrhea virus (PEDV) variants with large deletions in the spike (s) gene coexist with PEDV strains possessing an intact s gene in domestic pigs in Japan: a new disease situation. Plos One.

[2] Park JE, Cruz DJ, Shin HJ (2014). Clathrin- and serine proteases-dependent uptake of porcine epidemic diarrhea virus into Vero cells. Virus research.

[3] Luo X (2017). Tight junction protein occludin is a porcine epidemic diarrhea virus entry factor. J Virol.

[4] Li BX, Ge JW, Li YJ (2007). Porcine aminopeptidase N is a functional receptor for the PEDV coronavirus. Virology.

[5] Liu C (2015). Receptor usage and cell entry of porcine epidemic diarrhea coronavirus. J Virol.

[6] Shirato K (2016). Porcine aminopeptidase N is not a cellular receptor of porcine epidemic diarrhea virus, but promotes its infectivity via aminopeptidase activity. J Gen Virol.

[7] Gundersen GG (2002). Evolutionary conservation of microtubule-capture mechanisms. Nat Rev Mol Cell Biol.

[8] Dohner K, Nagel CH, Sodeik B (2005). Viral stop-and-go along microtubules: taking a ride with dynein and kinesins. Trends Microbiol.

[9] Dodding MP, Way M (2011). Coupling viruses to dynein and kinesin-1. Embo j.

[10] Hirokawa N (1998). Kinesin and dynein superfamily proteins and the mechanism of organelle transport. Science.

[11] Welte MA (2004). Bidirectional transport along microtubules. Curr Biol.

[12] Ross JL, Ali MY, Warshaw DM (2008). Cargo transport: molecular motors navigate a complex cytoskeleton. Curr Opin Cell Biol.

[13] McDonald D (2002). Visualization of the intracellular behavior of HIV in living cells. J Cell Biol.

[14] Suikkanen S (2003). Exploitation of microtubule cytoskeleton and dynein during parvoviral traffic toward the nucleus. J Virol.

[15] Seisenberger G (2001). Real-time single-molecule imaging of the infection pathway of an adeno-associated virus. Science.

[16] Mouland AJ (2001). RNA trafficking signals in human immunodeficiency virus type 1. Mol Cell Biol.

[17] Jouvenet N, Monaghan P, Way M, Wileman T (2004). Transport of African swine fever virus from assembly sites to the plasma membrane is dependent on microtubules and conventional kinesin. J Virol.

[18] Miyauchi K, Kim Y, Latinovic O, Morozov V, Melikyan GB (2009). HIV enters cells via endocytosis and dynamin-dependent fusion with endosomes. Cell.

[19] van der Schaar HM (2008). Dissecting the cell entry pathway of dengue virus by single-particle tracking in living cells. PLoS pathogens.

[20] Zeichhardt H, Otto MJ, McKinlay MA, Willingmann P, Habermehl KO (1987). Inhibition of poliovirus uncoating by disoxaril (WIN 51711). Virology.

[21] Brandenburg B (2007). Imaging poliovirus entry in live cells. PLoS biology.

[22] Tyagi S (2009). Imaging intracellular RNA distribution and dynamics in living cells. Nat Methods.

[23] Lakadamyali M, Rust MJ, Zhuang X (2004). Endocytosis of influenza viruses. Microbes Infect.

[24] van der Schaar HM (2007). Characterization of the early events in dengue virus cell entry by biochemical assays and single-virus tracking. J Virol.

[25] Lakadamyali M, Rust MJ, Babcock HP, Zhuang X (2003). Visualizing infection of individual influenza viruses. Proc Natl Acad Sci USA.

[26] Ayala Nunez NV, Wilschut J, Smit JM (2011). Monitoring virus entry into living cells using DiD-labeled dengue virus particles. Methods.

[27] Coller KE (2009). RNA interference and single particle tracking analysis of hepatitis C virus endocytosis. PLoS pathogens.

[28] Leary JF, Notter MF (1982). Kinetics of virus adsorption to single cells using fluorescent membrane probes and multiparameter flow cytometry. Cell Biophys.

[29] Lakadamyali M, Rust MJ, Zhuang X (2006). Ligands for clathrin-mediated endocytosis are differentially sorted into distinct populations of early endosomes. Cell.

[30] Hong ZY (2015). Clicking hydrazine and aldehyde: the way to labeling of viruses with quantum dots. ACS Nano.

[31] Huang LL, Xie HY (2014). Progress on the labeling and single-particle tracking technologies of viruses. Analyst.

[32] Liu SL (2012). Effectively and efficiently dissecting the infection of influenza virus by quantum-dot-based single-particle tracking. ACS Nano.

[33] Joo KI (2008). Site-specific labeling of enveloped viruses with quantum dots for single virus tracking. ACS Nano.

[34] Lv C (2016). Labeling viral envelope lipids with quantum dots by harnessing the biotinylated lipid-self-inserted cellular membrane. Biomaterials.

[35] Hao J, Huang LL, Zhang R, Wang HZ, Xie HY (2012). A mild and reliable method to label enveloped virus with quantum dots by copper-free click chemistry. Anal Chem.

[36] Reed LJ, Muench H (1938). A simple method of estimating fifty per cent endpoints. Am. j. hyg.

[37] Kopp M, Klupp BG, Granzow H, Fuchs W, Mettenleiter TC (2002). Identification and characterization of the pseudorabies virus tegument proteins UL46 and UL47: role for UL47 in virion morphogenesis in the cytoplasm. J Virol.

[38] Pelkmans L, Kartenbeck J, Helenius A (2001). Caveolar endocytosis of simian virus 40 reveals a new two-step vesicular-transport pathway to the ER. Nat Cell Biol.

[39] Ma Y (2016). Real-time imaging of single HIV-1 disassembly with multicolor viral particles. ACS Nano.

[40] Ruan G, Agrawal A, Marcus AI, Nie S (2007). Imaging and tracking of tat peptide-conjugated quantum dots in living cells: new insights into nanoparticle uptake, intracellular transport, and vesicle shedding. J Am Chem Soc.

[41] Liu SL (2011). Visualizing the endocytic and exocytic processes of wheat germ agglutinin by quantum dot-based single-particle tracking. Biomaterials.

[42] Sun EZ, Liu AA, Zhang ZL (2017). Real-time dissection of distinct dynamin-dependent endocytic routes of influenza a virus by quantum dot-based single-virus tracking. ACS Nano.

[43] Liu AA (2016). Simultaneous visualization of parental and progeny viruses by a capsid-specific HaloTag labeling strategy. ACS Nano.

[44] Zheng LL, Li CM, Zhen SJ, Li YF, Huang CZ (2017). A dynamic cell entry pathway of respiratory syncytial virus revealed by tracking the quantum dot-labeled single virus. Nanoscale.

[45] Marsh M, Helenius A (2006). Virus entry: open sesame. Cell.

[46] Cherry S, Perrimon N (2004). Entry is a rate-limiting step for viral infection in a Drosophila melanogaster model of pathogenesis. Nat Immunol.

[47] Leopold PL (2000). Dynein- and microtubule-mediated translocation of adenovirus serotype 5 occurs after endosomal lysis. Hum Gene Ther.

[48] Diefenbach RJ (2002). Herpes simplex virus tegument protein US11 interacts with conventional kinesin heavy chain. J Virol.

[49] Liu SL (2014). Three-dimensional tracking of Rab5- and Rab7-associated infection process of influenza virus. Small.

[50] Wang ZG (2014). Exploring sialic acid receptors-related infection behavior of avian influenza virus in human bronchial epithelial cells by single-particle tracking. Small.

[51] Greber UF, Way M (2006). A superhighway to virus infection. Cell.

[52] Liu SL (2014). Globally visualizing the microtubule-dependent transport behaviors of influenza virus in live cells. Anal Chem.

