# Supplementary material for: Real-time analysis of quantum dot labeled single porcine epidemic diarrhea virus moving along the microtubules using single particle tracking
Source: Sci Rep. 2019 Feb 4;9:1307. doi: 10.1038/s41598-018-37789-9 (PMC6362069; doi:10.1038/s41598-018-37789-9)
Supplement: Supplementary file 1 — Real-time analysis of quantum dot labeled single porcine epidemic diarrhea virus moving along the microtubules using single particle tracking [file 41598_2018_37789_MOESM1_ESM.pdf]

Supporting Information

Real-time analysis of quantum dot labeled single porcine epidemic diarrhea virus moving along the microtubules using single particle tracking

Wei Hou[a,†], Yangyang Li[a,†], Wenjie Kang[a], Xin Wang[a], Xuping Wu[b], Shouyu Wang[a,c], Fei Liu[a,*]

[a]Joint International Research Laboratory of Animal Health and Food Safety & Single Molecule Nanometry Laboratory (Sinmolab), Nanjing Agricultural University, Nanjing 210095, China.

[b]The Second Hospital of Nanjing Affiliated to Southeast University, Nanjing 210003, P.R. China.

[c]Computational Optics Laboratory, School of Science, Jiangnan University, Wuxi 214122, China.

[†]These two authors contributed equally to this work.

[*]Corresponding author: feiliu24@njau.edu.cn

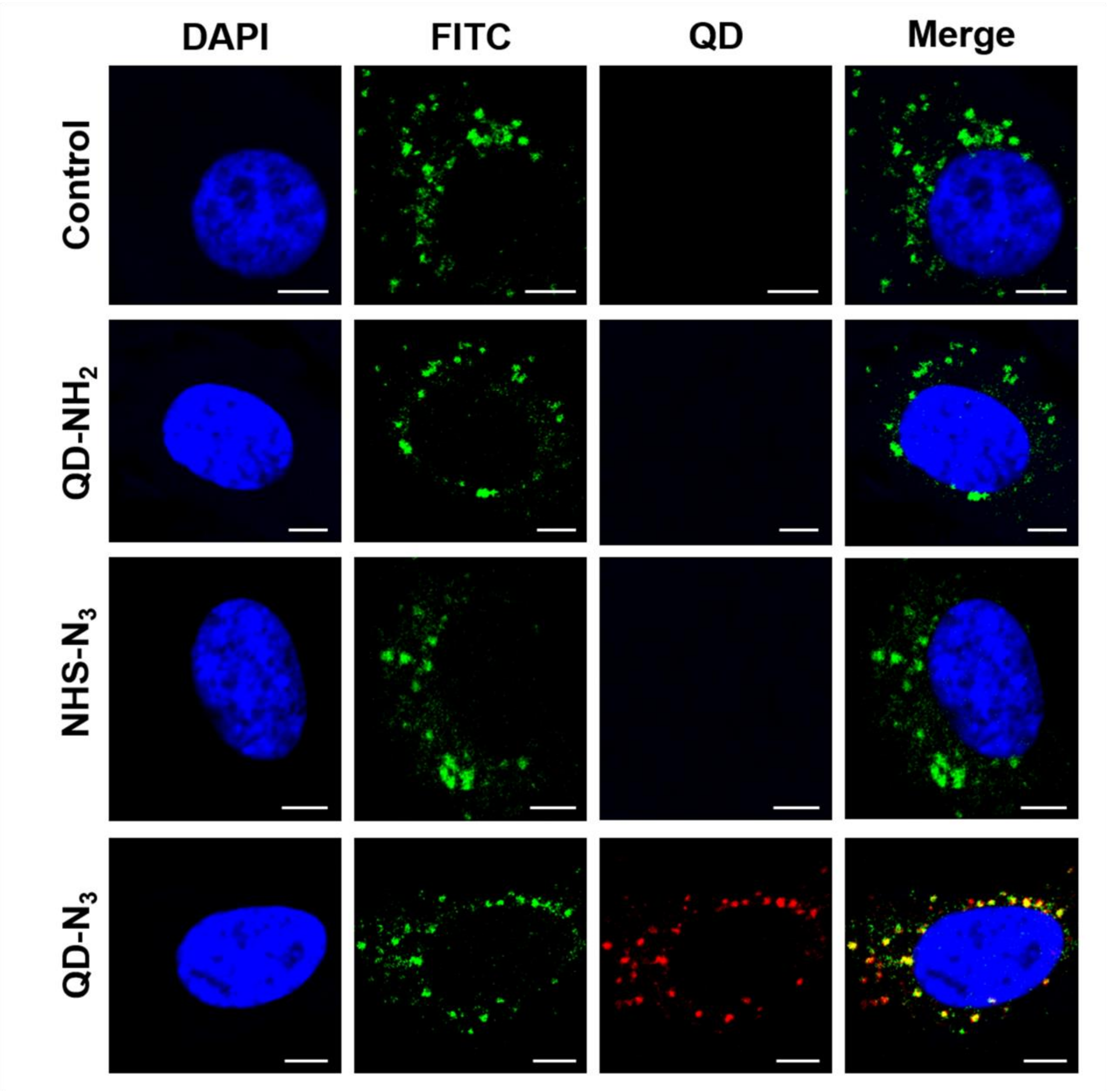

Figure S1. The characteristic of QD-N_3. The DBCO-virus was incubated with QD-NH_2, NHS-N_3 or QD-N_3 for 1 h at room temperature, respectively. Unreacted QD-NH_2, NHS-N_3 or QD-N_3 was removed by gel filtration on a NAP-10 column. Then, Vero cells were incubated with the as-prepared viruses at 4 ℃ for 30 min to allow virus attachment followed by incubation for 1 h at 37 ℃ with 5% CO_2. Afterwards cells were fixed and stained with the mouse monoclonal antibody against N protein and FITC labeled goat anti-mouse IgG polyclonal antibody (green), and the DBCO-virus without QD-NH_2, NHS-N_3 or QD-N_3 incubation served as the control (red, QD; blue, DAPI labeled cell nucleic acid). Scale bars indicate 5 μm of regular views.

Supporting Videos

Video S1: Viruses moving along the microtubules in the cells.

Video S2: Enlarged view of one single virus moving along the microtubules in the cells selected from Video S1.

Video S3: Virus moving along the microtubules near the CM.

Video S4: Virus moving along the microtubules in the CC.

Video S5: Virus moving along the microtubules near the MTOC.

Video S6 and S7: Virus moving along the microtubules with a return movement in Vero cells.
